# Mucosal Immune Response to Feline Enteric Coronavirus Infection

**DOI:** 10.3390/v11100906

**Published:** 2019-09-27

**Authors:** Morgan Pearson, Alora LaVoy, Samantha Evans, Allison Vilander, Craig Webb, Barbara Graham, Esther Musselman, Jonathan LeCureux, Sue VandeWoude, Gregg A. Dean

**Affiliations:** 1 Department of Microbiology, Immunology and Pathology, Colorado State University, Fort Collins, CO 80523, USA; morganepearson@gmail.com (M.P.); Alora.lavoy@colostate.edu (A.L.); evans.2608@osu.edu (S.E.); Allison.Vilander@colostate.edu (A.V.); Barb.Graham@colostate.edu (B.G.); esther.musselman@gmail.com (E.M.); jslecureux@gmail.com (J.L.); sue.vandewoude@colostate.edu (S.V.); 2 Department of Clinical Sciences, Colorado State University, Fort Collins, CO 80523, USA; Craig.Webb@ColoState.EDU

**Keywords:** Coronavirus, feline enteric coronavirus, mucosal immunity, feline infectious peritonitis

## Abstract

Feline infectious peritonitis is a devastating, fatal disease of domestic cats caused by a pathogenic mutant virus derived from the ubiquitous feline enteric coronavirus (FECV). Infection by FECV is generally subclinical, and little is known about the mucosal immune response that controls and eliminates the virus. We investigated the mucosal immune response against FECV in an endemically infected breeding colony over a seven-month period. Thirty-three cats were grouped according to FECV seropositivity and fecal virus shedding into naïve/immunologically quiescent, convalescent and actively infected groups. Blood, fecal samples and colon biopsies were collected to assess the mucosal and systemic immunologic and virologic profile. Results showed that cats with active FECV infections have strong systemic IgG and mucosal IgA responses that wane after virus clearance. Significant FECV-specific mucosal T cell IFNγ responses were not detected in any of the three groups. A shift toward an inflammatory state in the mucosa was suggested by increased IL17:FoxP3 expression. However, no histologic abnormalities were observed, and no shifts in lymphocyte subpopulation phenotype or proliferation were noted. Together, the results suggest that control of FECV is mediated by humoral mucosal and systemic responses and that perturbations in the primary reservoir organ (colon) are minimal.

## 1. Introduction

Feline infectious peritonitis remains intractable with regard to diagnosis, treatment and prevention (reviewed in [1,2]). It is generally accepted that feline infectious peritonitis virus (FIPV) emerges in individual cats due to the mutation of replicating feline enteric coronavirus (FECV) [2]. FECV is mucosally transmitted, replicates to the greatest levels in the intestinal tract, and most commonly persists in the colonic epithelium [3,4]. While some cats are able to control FECV replication and eliminate the infection, others remain infected and are intermittent or persistent virus shedders [5,6,7]. Additionally, immunity to FECV is not life-long, so reinfection can occur, with FECV continually churning in cat populations, particularly within shelters or breeding colonies through cycles of repeated exposure, infection, and shedding. Within FECV-infected cats, the combination of high virus replication and high error rate in RNA replication results in innumerable mutations. If a mutation occurs that allows the virus to escape immune surveillance and efficiently infect and replicate in monocytes, it can then systemically spread, and fatal FIP may occur [2]. Vaccine strategies have targeted the mutant FIPV, but this approach has been ineffective and, in some cases, has resulted in accelerated disease as a result of the antibody-dependent enhancement of infection (reviewed in [2,8]).

To date, there have been few attempts to control the source of FIP-causing mutant virus: FECV replication. Excellent epidemiologic studies have characterized the shedding and transmission of FECV within populations. FECV circulates within cat populations and is epidemiologically characterized by repeated infections, persistent shedding, and carrier states [3,5,6]. FECV is highly contagious, as shown by Pedersen et al. where 28/50 (56%) FECV-negative cats were infected within one week after introduction to an animal shelter [9]. In addition, at the time of entering the shelter, 33% of cats were already shedding FECV, and their level of shedding increased 10 to 1,000,000 fold during the first week, which was likely secondary to the stress associated with shelter admission. The high incidence of infection combined with massive virus replication greatly enhances the likelihood that a mutant virus will emerge.

While the immunological response to FIPV has been extensively studied, less is known about the response to FECV, particularly in the intestinal mucosa. Here, we studied a colony of 33 co-housed cats with endemic FECV for seven months. Blood, feces and colon biopsies were collected to establish the relationship between virus control and the mucosal and systemic immune response. Results showed that mucosal IgA and systemic IgG are associated with a clearance of virus infection, while no cell-mediated immune response was detected. The humoral response waned rapidly after the virus was cleared, and only minimal structural or functional perturbations could be demonstrated in the colon. These results suggest that the control of FECV at the intestinal mucosa might be achievable by a vaccine-induced humoral response with the goal of disrupting the endemic persistence of FECV in cat populations to reduce the chance of FIPV emergence in individuals.

## 2. Materials and Methods

### 2.1. Animals

Cats were cared for in accordance with the Association for the Assessment of Laboratory Animal Care standards and with approval from the Colorado State University Institutional Animal Care and Use Committee (protocol 16-6390A, approved 11 July, 2017). Thirty-three domestic shorthair cats maintained in a specific pathogen-free (SPF) breeding colony at Colorado State University were used in the study. Cats ranged from 1 to 9 years of age and included 22 intact females, 10 neutered males, and 1 intact male. The colony was known to have circulating feline coronavirus (FCoV) based on seropositivity and the sporadic occurrence of FIP since the establishment of the colony in the 1980s. The genome of the circulating virus was sequenced and shown to be serotype 1. Cats were the offspring of colony matings, queens were individually housed prior to parturition, and offspring were introduced into the colony when they reached breeding age. Cats were group housed, with the exception of intact males, which were housed together in their own run in the same room as the other cats. 

### 2.2. Sample Collection and Processing

The day of colonoscopy was designated as time 0. Fecal samples and blood were collected at experimental months −2, 0, 1, 2, 3, 4 and 5. Fecal samples, collected from the litter boxes of cats temporarily housed individually, were stored at −80 °C. Whole blood for peripheral blood mononuclear cell (PBMC) and plasma isolation, complete blood counts (CBC), and leukocyte differentials were collected into Vacutainer tubes containing ethylenediaminetetraacetic acid (BD, Franklin Lakes, NJ, USA); blood for serum chemistry was collected in serum Vacutainer tubes. PBMC were isolated by the centrifugation of blood over Histopaque-1077 (MilliporeSigma, St. Louis, MO, USA), and pellets were stored at −80 °C [10,11]. Plasma was isolated by centrifugation at 2200× *g* for 15 min, and aliquots were submitted for a full chemistry panel or frozen at −80 °C. CBC, leukocyte differentials, and a full serum chemistry panel were performed by the Colorado State University Clinical Pathology Laboratory. The colonoscopy and biopsy (18−20 2-mm pinch biopsies) were performed at month 0 under general gas anesthesia after an overnight fast. Colon biopsy tissues were collected in 10% formalin for histology and immunohistochemistry (IHC), RNAlater for RNA extraction, or RPMI medium with 1× penicillin–streptomycin (GE Healthcare Life Sciences, Pittsburgh, PA, USA) and 10 µg/mL gentamicin (MilliporeSigma) for lymphocyte isolation.

### 2.3. Mucosal Lymphocyte Isolation

Colon biopsy samples were processed using modifications of a previously described protocol [12]. Briefly, endoscopic biopsies were digested in 2 mL of a digestion medium consisting of RPMI without l-glutamine (Corning, Tewksbury, MA, USA) with added 1× penicillin–streptomycin (GE Healthcare Life Sciences), 50 µg/mL of Liberase DL (MilliporeSigma), and 1 mg/mL of DNase I, grade II (MilliporeSigma) for 30 min at 37 °C. Tissue was then passed through a sterile 16-gauge needle 10–15 times, then through a 100 µm EasyStrainer (Greiner Bio-One, Monroe, NC, USA). Large pieces of undigested tissue were returned to 37 °C for 30 min with an additional 2 mL of fresh digestion medium. This process was repeated twice for a total of 3 digestion steps. All three cell suspensions were combined and passed through 70 and 40 µm EasyStrainers (Greiner Bio-One), then washed with LBT medium (RPMI medium supplemented with 10% fetal bovine serum, 15 mM of HEPES, 1 mM of sodium pyruvate, 4 mM of l-glutamine, 10 IU of penicillin/mL, and 10 μg of streptomycin/mL) and filtered into a Falcon tube with a 35 µm cell strainer cap (Corning). Live nucleated cells were counted using ViaStain AOPI staining solution in a Cellometer Auto 2000 (Nexcelom Bioscience, Lawrence, MA, USA), according to the manufacturer’s directions.

### 2.4. Immunophenotyping

Approximately 200,000 mucosal lymphocytes were used for the flow cytometric analysis. Standard staining protocols were used, as previously described [13]. Briefly, cells were blocked with 1% protease-free bovine serum albumin (Equitech-Bio, Kerrville, TX) and were then stained with the following antibodies: Mouse anti-cat CD45 (clone 30.7.9, Dean et al., unpublished results), goat anti-mouse IgG2A conjugated to APC-Cy7 (SouthernBiotech, Birmingham, AL, USA), mouse anti-cat CD4 conjugated to FITC (clone 3-4F4, SouthernBiotech), mouse anti-cat CD8 conjugated to APC (clone 3.357, Clinical Immunology Laboratory, NCSU, Raleigh, NC, USA), and rat anti-mouse B220 conjugated to PerCP (clone RA3-6B2, BioLegend, San Diego, CA, USA). Cells were then washed and treated with Fixation/Permeabilization Solution Kit (BD Biosciences, San Jose, CA, USA). After washing, cells were stained intracellularly with mouse anti-human Ki-67 conjugated to PE (BD Biosciences). At least 50,000 cells were analyzed using a Gallios flow cytometer (Beckman Coulter, Indianapolis, IN, USA). Data analysis was completed using FlowJo Software, version 10 (Tree Star, Ashland, OR, USA).

### 2.5. FCoV Specific IgG in Plasma

FCoV-specific IgG was determined using a commercially available kit (Feline Infectious Peritonitis Virus Antibody Test Kit, IVD Technologies, Santa Ana, CA, USA) according to the manufacturer’s instructions.

### 2.6. Fecal Total IgA ELISA

Fecal samples were thawed on ice, a portion was transferred into a tube and weighed, 10% goat serum (Equitech-Bio) and 2% ProteaseArrest (G-Biosciences, St. Louis, MO, USA) in PBS was added (1 mL per 100 mg feces); samples were then homogenized for 1 min at 6.5 m/s in a FastPrep 24 instrument (MP Biomedicals, Irvine, CA, USA), centrifuged 5 min at 12,000× *g*, and aliquots of the supernatant were stored at −80 °C. Microlon high binding 96-well plates (Greiner Bio-One) were coated overnight at 4 °C with 100 µL of 2 µg/mL goat anti-cat IgA (Bethyl, Montgomery, TX, USA) in carbonate/bicarbonate buffer pH 9.5, and then the coating solution was replaced with 5% nonfat dry milk in coating buffer for 1 hour at 37 °C. After 5 washes with 0.05% Tween-20 in phosphate buffered saline (PBST, used for all ELISA and ELISPOT wash steps), 100 µL/well fecal supernatant or feline reference serum (Bethyl) serially diluted in PBST with 10% nonfat dry milk and 20% goat serum was added in duplicate and incubated at 37 °C for 1 h. Plates were washed 5 times, and then 100 µL/well of 0.5 µg/mL goat anti-cat IgA (Bethyl) was added for 1 h at 37 °C. After 6 washes, SureBlue Reserve TMB 1-Component Microwell Peroxidase Substrate (SeraCare, Milford, MA, USA) was added for 10 min at room temperature (RT), and then development was stopped with 1 N HCl and absorbance was measured (450 nm, minus background measured at 570 nm) in a Synergy 2 microplate reader with Gen5 software version 1.11.5 (BioTek, Winooski, VT, USA). A standard curve was generated from reference serum wells using Gen5. 

### 2.7. FCoV-Specific IgA ELISA 

Microlon high binding 96-well plates (Greiner Bio-One) were coated overnight at 4 °C with 100 µL/well of 15 µg/mL feline infectious peritonitis virus antigen (IVD Technologies) in PBS pH 7.4, and then the coating solution was replaced with 5% nonfat dry milk in PBS for 2 h at RT. Plates were washed 4 times with PBST with a 5 min soak between washes 3 and 4. Fecal extracts (prepared as for the total IgA ELISA) and a positive reference sample were serially diluted in 5% nonfat dry milk and 5% normal goat serum in PBST, and 100 µL was added per well, in duplicate. Plates were incubated at 37 °C for 3 h, washed 6 times with a 5 min soak between washes 3 and 4, and 100 µL/well of 0.5 µg/mL goat anti-cat IgA (Bethyl) was added for 1 h at 37 °C. After 6 washes, SureBlue Reserve TMB Substrate was added for 10 min at RT, then development was stopped with 1 N HCl, and absorbance was measured (450 nm, minus background measured at 570 nm). 

### 2.8. RNA Extraction from Fecal Samples

Fecal samples were thawed on ice, a portion was transferred into a tube and weighed, sterile saline was added (1 mL per 100 mg feces), and then samples were homogenized for 1 min at 6.5 m/sec in a FastPrep 24 instrument (MP Biomedicals, Irvine, CA, USA). After centrifugation (5 min at 12,000× *g*), RNA was extracted from the supernatant with QIAamp Viral RNA mini kit (QIAGEN, Germantown, MD, USA) according to the manufacturer’s protocol with 4 volumes supernatant, Buffer AVL, and 100% ethanol. RNA was eluted with 40 µL of AVE buffer, 2% murine RNase inhibitor (New England Biolabs, Ipswich, CA, USA) was added, and RNA was stored at −80 °C. A buffer-only extraction was included in each extraction batch. 

### 2.9. RNA Extraction from Colon Tissue and PBMC

RNA was extracted from colon biopsy tissue using the RNeasy Powerlyzer Tissue and Cells kit per manufacturer’s protocol (QIAGEN). Briefly, tissues were homogenized with two 45-second cycles at 5.5 m/s in the FastPrep 24, and an on-column treatment with RNase-free DNase was included. RNA was extracted from PBMC using the RNeasy Mini Kit, including QIAshredder and RNase-free DNase treatments (QIAGEN). An RNase inhibitor was added to eluted RNA before storage at −80 °C. RNA was quantified with the Qubit RNA BR assay kit (Thermo Fisher Scientific, Waltham MA, USA), and quality was assessed by spectrophotometry and by a RNA High Sensitivity ScreenTape assay (Agilent, Santa Clara, CA, USA). A buffer-only extraction was included in each tissue or PBMC extraction batch. 

### 2.10. FCoV Real-Time RT-PCR

RNA was analyzed for presence of FCoV RNA by a real-time RT-PCR (RT-PCR) amplification of a well-conserved region of the membrane and nucleocapsid genes using primers (400 nM each) and probe sequences (200 nM, 5′ 6-FAM/ZEN/3′IB^®^FQ-labeled; Integrated DNA Technologies, Coralville, IA, USA), as previously described in [14] with a Luna Universal Probe One-Step RT-PCR reagent (New England Biolabs). Samples (5 µL or 0.5 µL of fecal RNA, or 100 ng of colon or PBMC RNA) were run in triplicate 20 µL reactions in white 96-well PCR plates (Bio-Rad, Hercules, CA, USA) using a Bio-Rad CFX96 instrument and the following cycling conditions: Reverse transcription (55 °C, 10 min), initial denaturation (95 °C, 1 min), 45 amplification cycles (95 °C 10 s, 60 °C 30 s), and final extension (72 °C, 10 min). Serial dilutions of FCoV-positive RNA were included on each plate for quality control. No FCoV RNA was detected in the buffer-only extraction controls or in the no-template controls included on each plate.

### 2.11. IL17 and FoxP3 Real-Time RT-PCR

RT-PCR reactions were carried out as for FCoV, with 100 ng of colon RNA. FoxP3 primer and probe sequences were previously published [15]. IL17 primers: Forward TGGCTCCTGGGACAACTTC, reverse TCCTCGGTAGTTGGGCATCC, and probe TCCCATCACTGCTGCTGCTGCTCT. All probes were 5’ 6-FAM/ZEN/3′IB^®^FQ-labeled (IDT). Additional controls included no-RT reactions for each sample with each primer/probe set and a real-time internal control RNA reaction (Primerdesign Ltd, Plymouth Meeting, PA, USA) for each sample for inhibitor analysis. No RT-PCR inhibition was detected. 

### 2.12. Total and FCoV-Specific IgA ELISPOT

MultiScreen-IP 96-well plates (MAIPSWU10; MilliporeSigma) were pre-wet with 70% ethanol, washed 5 times with sterile PBS, and then incubated overnight at 4 °C with 100 µL of goat anti-cat IgA (Bethyl) diluted to 15 µg/mL in PBS or with PBS without antibody as a negative control. Plates were washed as before and blocked for 2 h at RT with the LBT medium, then 5000 cells/well (total IgA, 3 each coated with IgA or PBS) or 25,000 cells/well (FCoV IgA, 10 IgA-coated wells and 5 PBS wells) in LBT were added and incubated 4 h at 37 °C, 5% CO_2_. Plates were washed 3 times with PBS and 3 times with PBST before the addition of 100 µL/well 0.22 µm-filtered detection reagent diluted to 1 µg/mL in 0.5% fetal bovine serum/PBS and overnight incubation at 4 °C. For total IgA, the detection reagent was 1 µg/mL goat anti-cat IgA-HRP (Bethyl). For FCoV IgA, the detection reagent was 1 µg/mL FIPV antigen (IVD) biotinylated using a ChromaLink Biotin Labeling Kit (TriLink Biotechnologies, San Diego, CA, USA); after incubation, plates were washed 4 times with PBST and then incubated with 100 µL/well of 1 µg/mL Pierce streptavidin-HRP (Thermo Fisher Scientific) for 1 h at RT. For both assays, plates were then washed 3 times with PBST and 3 times with PBS, and spots were developed using 0.45 µm-filtered TMB Substrate for ELISPOT (Mabtech, Inc, Cincinnati, OH) followed by 6 washes with ultrapure water. After drying, plates were scanned with an ImmunoSpot S5 Analyzer (CTL, Cincinnati, OH, USA) and spots were counted using ImmunoSpot software version 7 (CTL).

### 2.13. IFNγ ELISPOT

Capture and detection antibodies from the feline IFNγ Development Module (R&D Systems) were used with MultiScreen-IP 96-well plates (MAIPSWU10; MilliporeSigma) to quantify IFNγ-producing mucosal lymphocytes after stimulation with phorbol 12-myristate 13-acetate (PMA, 50 ng/mL; LC Laboratories, Woburn, MA) and ionomycin (300 ng/mL; LC Laboratories) or with FIPV antigen (60 µg/mL; IVD Technologies, Santa Ana, CA, USA) as previously described [16]. The protocol was modified with the use of 2.5 × 10^4^ cells/well (PMA/ionomycin) or 2 × 10^5^ cells/well (FIPV antigen) incubated for 40 h at 37 °C, 5% CO_2_. Plates were scanned with an ImmunoSpot S5 Analyzer, and analysis was performed by CTL. 

### 2.14. Histology and FCoV Immunohistochemistry

Formalin-fixed tissues were processed and stained with hematoxylin and eosin or for FCoV antigen by the Colorado State University Veterinary Diagnostic Laboratories. Briefly, the FCoV immunohistochemistry was performed using a mouse anti-feline coronavirus primary (clone FIPV3-70, Bio-Rad) and PowerVision poly-AP anti-mouse IgG secondary (Leica Microsystems Inc., Buffalo Grove IL) by heat-induced epitope retrieval on a Leica Bond-III IHC automated stainer using a Bond Epitope Retrieval Solution 2^a^ for 20 min. Labeling was performed on an automated staining platform. Fast Red was used as the chromogen, and slides were counterstained with hematoxylin.

### 2.15. Statistical Analysis

All analyses were performed in the open-source program R version 3.5.1 [17]. The log-transformed values of the variables were evaluated separately using linear models. Variables with zero values had a small constant added prior to log-transformation (1 or 0.01, based on the range of values). Residuals for all models were tested for normality and homoskedasticity, and none were found to violate those assumptions. Group means and confidence intervals were extracted from the models using the package emmeans version 1.2.3 [18]. Data were handled with the package dplyr version 0.7.4 [19], and plots were created using the package ggplot2 version 2.2.1 [20]. 

## 3. Results

### 3.1. Plasma IgG and Fecal Real-Time RT-PCR 

The FCoV infection status of each cat included in this study was determined by an ELISA for FCoV-specific plasma IgG and RT-PCR for fecal virus RNA. Based on these data, cats were grouped for further analysis (Table 1). Group 1 cats (*N* = 18; average age, 3.1 yrs.; range, 2.2–6.5 yrs) were FCoV seronegative and virus negative throughout the study, but given that these cats were gang housed, they were most likely exposed to FCoV and possibly infected at some point prior to the study period. Group 2 cats (*N* = 6; average age, 5.1 yrs.; range, 4.2–5.8 yrs) were seropositive at the time of colonoscopy but never tested positive for fecal or tissue (colon biopsy or blood) virus. Group 3 cats (*N* = 9; average age, 3.8 yrs.; range, 2.0–6.6 yrs) were seropositive and were also fecal virus positive at timepoint −2 and/or 0 months. Eight of the nine Group 3 cats controlled virus shedding within three months from the start of the study. The cat with uncontrolled virus shedding remained fecal virus positive throughout the study period, and its colon biopsy was also virus positive. The serological status and RT-PCR results for each group are shown in Table 1. These groups were anticipated to represent different immunological states that might identify correlates of protection against FECV. Group 1 cats represented an immune naïve or immune quiescent state, while Group 2 cats represented the convalescent phase of the immune response. It was fortuitous to collect colon biopsies on eight cats in Group 3 very near the time that virus replication was controlled, as this presumably provided a snapshot of a successful mucosal immune response to the virus. The average time of observed virus shedding in cats that ultimately controlled virus replication was 2.6 months, and the average time of seropositivity after virus replication was controlled was 2.5 months. These data reveal less about the virus infection kinetics, since this was a study of naturally infected cats, and the actual time of infection was not known. More can be concluded about the systemic immune response, which waned rapidly after virus was cleared. Of the cats that were seropositive at the time of colon biopsy (Groups 2 and 3), 11 of the 15 were seronegative by the end of the study. 

### 3.2. CBC, Serum Chemistry, PBMC RT-PCR

On the day of colonoscopy and biopsy, a complete blood count and full serum chemistry panel was performed. No abnormalities were identified for any individual animal, and no differences were observed between groups (Table S1). PBMC were analyzed for FCoV by RT-PCR, and all were negative. These results are consistent with the subclinical nature of FECV infection and sporadic viremia.

### 3.3. Fecal IgA

**Figure 1:**
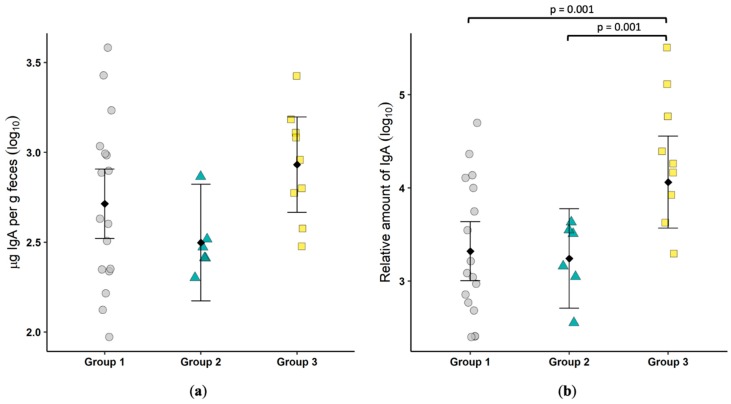
Feline coronavirus (FCoV)-specific IgA is increased in cats shedding virus in feces. Total fecal IgA, shown as µg IgA per gram of feces (**a**) and FCoV-specific fecal IgA, shown as relative amount (**b**) were determined by an ELISA. Each symbol represents a single cat. Bars show the mean and 95% confidence interval. Linear models were used to compare group means, and *p* values are shown when ≤0.05.

Total fecal IgA was measured by an ELISA. There was a trend for Group 3 cats to have higher levels, but the difference did not reach significance (Figure 1a). FCoV-specific fecal IgA was significantly higher (*p* = 0.001) in cats actively shedding the virus as compared to either Group 1 or Group 2 (Figure 1b). This result suggests a role for mucosal IgA in the control and clearance of FECV infection. 

### 3.4. Colonic Biopsy, Histopathology, FCoV Immunohistochemistry, Tissue RT-PCR, and Flow Cytometry

Colonic pinch biopsies were collected for histopathology and were stained for FCoV antigen by immunohistochemistry. No histopathological abnormalities were noted, and no conclusive positive staining for FCoV antigen was observed for any cat. Positive and negative control tissues stained as expected. Tissue-associated virus was detected by RT-PCR in three of nine cats in Group 3; these cats were also positive for fecal virus (Table 1).

Lymphocytes were isolated from colonic pinch biopsies for phenotypic and proliferation analysis. The percentages of CD4+ T cells, CD8+ T cells and B cells were determined, and the percentage of each population that was Ki-67+ was measured to assess proliferation (Table 2). The lymphocytes from the biopsies represented both intraepithelial lymphocytes (IEL) and lamina propria lymphocytes (LPL). LPL are more numerous and are typically a mix of CD4+ and CD8+, while IEL are predominantly CD8+ in cats, mice, macaques, and humans [12,21,22,23,24]. There were no significant differences between the groups, indicating active FECV replication in Group 3 cats did not result in phenotypic shifts or increased lymphocyte proliferation in the colonic mucosa. Because there were no differences between groups, percentages were combined (*N* = 33) to provide reference ranges for future studies that examine colonic biopsies, since such an analysis has not been previously reported. 

### 3.5. IgA ELISPOT

**Figure 2:**
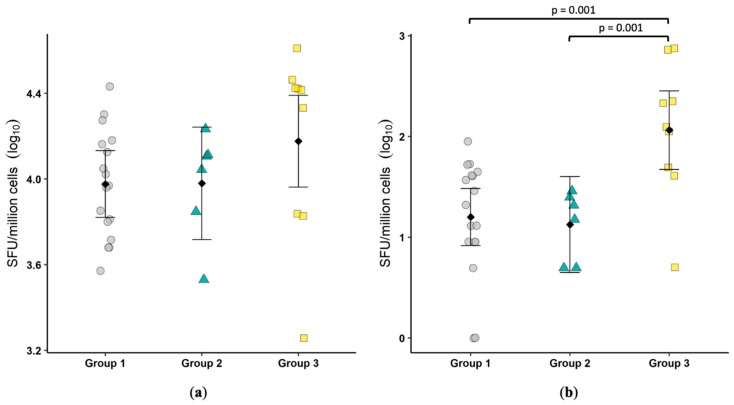
FCoV-specific IgA-secreting cells are increased in cats shedding virus in feces. The number of IgA-secreting cells (spot forming units, SFU) (**a**) and the number of FCoV-specific IgA-secreting cells (**b**) were determined by ELISPOT. Each symbol represents a single cat. Bars show the mean and 95% confidence interval. Linear models were used to compare group means, and *p* values are shown when ≤0.05. One was added to the number of FCoV-specific IgA-secreting cells prior to log transformation.

ELISPOT assays were undertaken on the lymphocytes isolated from the colonic biopsies to determine the total number of IgA-producing cells and the number of FCoV-specific IgA-producing cells. Similar to fecal IgA, no significant difference was observed in the number of IgA-producing cells (Figure 2a). The number of FCoV-specific IgA-producing cells was significantly greater (*p* = 0.001) in Group 3 as compared to either Group 1 or Group 2 (Figure 2b). As with fecal IgA results, it appears FECV replication induces a mucosal humoral response that may be important in limiting virus replication. 

### 3.6. Colon IFNγ ELISPOT and IL17:FoxP3 RT-PCR

**Figure 3:**
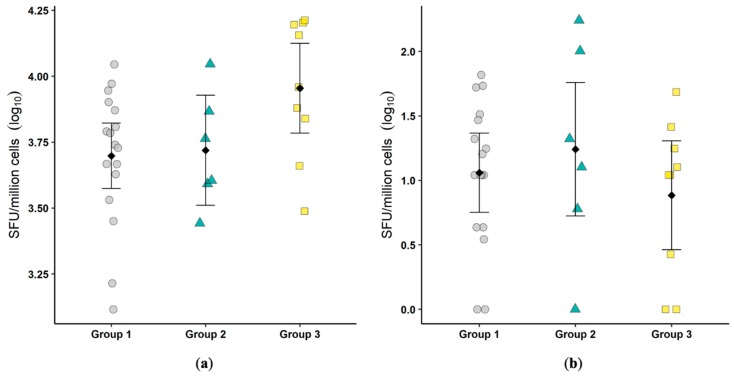
Infection by feline enteric coronavirus (FECV) did not result in an increase in total IFNγ-producing cells or FCoV-specific IFNγ-producing cells. The total number of IFNγ-secreting cells after phorbol 12-myristate 13-acetate (PMA)/ionomycin stimulation (spot forming units, SFU) (**a**) and the number of FCoV-specific IFNγ-secreting cells (**b**) were determined by ELISPOT. Each symbol represents a single cat. Bars show the mean and 95% confidence interval. Linear models were used to compare group means, and *p* values are shown when ≤0.05. One was added to the number of FCoV-specific IFNγ-secreting cells prior to log transformation.

The total number of colonic lymphocytes with the potential to produce IFNγ was determined by stimulation with PMA/ionomycin, and the total number of FCoV-specific IFNγ-producing cells was determined by re-stimulation with viral antigen. There were no significant differences between groups in the IFNγ-producing capacity or in the antigen-specific production (Figure 3). These results suggest that the T cell-mediated control of FECV infection is unlikely to play a role in the colonic mucosa.

**Figure 4:**
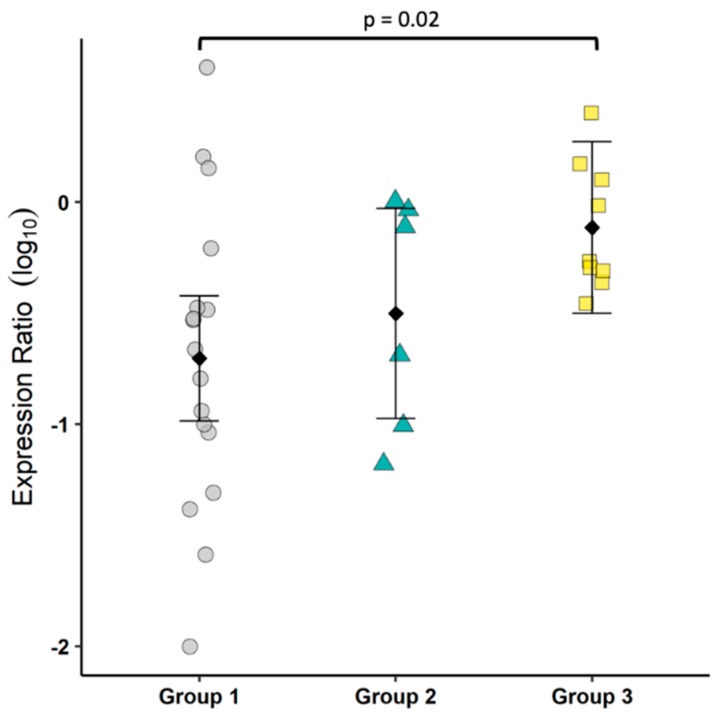
FECV infection induced an inflammatory bias, as indicated by IL17:FoxP3. The ratio of IL17 to FoxP3 transcripts was determined by real-time RT-PCR. Each symbol represents a single cat. Bars show the mean and 95% confidence interval. Linear models were used to compare group means, and *p* values are shown when ≤0.05. Prior to log transformation, 0.01 was added to the expression ratio.

As a means to assess the state of T cell balance between inflammation and regulation, the transcriptional ratio of IL17 and FoxP3 was determined by RT-PCR. Group 3 cats had a significantly higher ratio as compared to Group 1, indicating an overall tendency toward inflammation (Figure 4). 

## 4. Discussion

Feline coronavirus infection presents a virological and immunological conundrum. How does a relatively innocuous enteric pathogen that induces a modest immune response evolve into an uncontrollable systemic pathogen that drives a destructive immunological storm? The well-supported hypothesis is that the emergence of a viral mutant with an expanded host cell range permits the infection and the efficient replication in monocytes/macrophages, as well as the systemic the spread of virus that drives immunopathological pathways including lymphocyte apoptosis, antibody complex deposition, and granulomatous inflammation [25,26,27]. While the dramatic immune response associated with FIP has been extensively studied, much less is known about the mucosal immune response against FECV or how the successful control of FECV limits viral replication and enteric pathology (reviewed in [2]). 

We took advantage of a closed colony of SPF cats with endemic serotype I FCoV to characterize immune responses associated with virus control. Since the colon is the primary reservoir and site of replication for FECV, colon biopsies were both logical and convenient for mucosal immune response evaluation [3,4]. The distribution of enrolled cats, based on the combination of serological and fecal virus status, was similar to previous reports and allowed for a direct comparison of naïve/immune quiescent, convalescent, and actively infected cats [28]. Fortuitously, eight of the nine cats positive for fecal virus shedding controlled replication at or shortly after colonic biopsy. Thus, the immune profile observed in the tissue might provide correlates of protection against FECV. These cats had significantly elevated mucosal IgA along with systemic IgG against FCoV, but they had no measurable mucosal IFNγ T cell response. Based on these results, it appears that FECV control is mediated by antibody responses. 

FCoV-specific IgA was significantly elevated in cats actively shedding virus, as measured by a fecal ELISA and by the quantification of IgA-producing cells in the colonic mucosa. This suggests that secretory IgA (SIgA) may play a key role in preventing immunopathogenesis in FECV infections. SIgA is the most abundantly produced antibody isotype in the body and is highly resistant to proteolysis, engendering stability in the harsh environment of the intestinal tract [29]. SIgA is mucophilic and accumulates at greater concentration in the mucus layer covering the intestinal epithelium; therefore, it stands as the first adaptive immune defense against mucosal pathogens [30]. The effector functions of SIgA include virus neutralization and immune exclusion in the intestinal lumen. The intracellular inactivation of viruses and the removal of antigens from the lamina propria by IgA has also been demonstrated (reviewed in [31]). Additionally, in contrast to IgG, IgA does not activate complement, and this prevents inflammation and tissue damage during infections (reviewed in [32]). Though further studies are needed, a general lack of immune activation (as was measured in this study) combined with the effective control of viral replication suggests a key role for SIgA in subclinical FECV infection.

FECV infection results in remarkably few clinical or immunological perturbations despite astounding levels of virus replication [9]. We observed no abnormalities in the serum chemistry profile or CBC of Group 3 cats. Likewise, no cell-associated virus was detected in the blood, even though systemic dissemination during FECV infection has been well described [3]. It may be that we missed the window of viremia in these cats that were on the verge of controlling replication in the gastrointestinal tract [33]. Similarly, no FCoV antigen was demonstrated in the colonic biopsies and only three of the nine cats were positive for viral RNA in the biopsy tissue. This also suggests a waning infection and perhaps a low sensitivity of the assays given the very small amount of tissue (a single 2 mm biopsy) that was available for analysis. No differences were noted in mucosal lymphocyte phenotype percentages or proliferation between groups of cats, and this was consistent with a lack of inflammation on histology. Colonic inflammation can be multifocal, and it is possible that the colonic biopsies evaluated were not representative of the inflammatory state elsewhere in the colon. These features confirm the overall picture of a relatively benign enteric viral infection despite robust viral replication. 

To assess cell mediated immune responses, we measured constitutive and FCoV-specific IFNγ production. No differences were observed between groups, suggesting cellular sources of IFNγ including NK cells, NKT cells and T cells were not over-represented in the colonic mucosa of FECV-shedding cats. Furthermore, the lack of difference when cells were stimulated with FCoV antigen suggests a lack of a cytotoxic T cell adaptive response. The small number of colonic lymphocytes available for immunophenotyping limited the analysis, but the relatively low number of CD8+ cells was surprising. We previously reported the phenotype of intraepithelial and lamina propria lymphocytes (IEL and LPL, respectively) in the small intestine of cats [12]. While small intestine IEL were predominantly CD8+, the majority of LPL were CD4+ T cells. The method used here harvested IEL and LPL together and the phenotype of colonic lymphocytes has not been previously reported in cats. In mice and humans, CD4+ T cells predominate in the IEL and LPL of the colon [24]. Despite this dominance of CD4+ over CD8+ T cells in mice and humans, the very low percentage of CD8+ T cells in the colonic mucosa of cats (<5%) seems unusual. It could be an artifact of tissue processing or antibody selection; however, we used methods and reagents previously validated for the analysis of lymphocytes in the small intestine [12]. Further work is required to confirm these results and to functionally characterize feline colonic lymphocytes. 

To gain some understanding of the T cell bias in the colonic mucosa, we measured the mRNA of IL17 as an indicator of T cell inflammation and FoxP3 as an indicator of T cell negative regulation. Our results showed an increase in mucosal IL17:FoxP3 mRNA, suggesting a modest shift toward an inflammatory environment. There are many caveats to this approach as a proxy for the evaluation of Th17 and regulatory T cells (Treg), but the small tissue sample size and lack of feline-specific reagents to identify Th17 at the start of the study limited the options. Interestingly, a cross-reactive IL17A antibody has recently been reported and was used to assess FIP-associated uveitis [34]. Along with existing reagents and protocols to evaluate feline Treg by flow cytometry, future studies can more directly assess the relative presence of Th17 and Treg [15]. 

It has been hypothesized that a failed T cell response distinguishes between disease resistance and progressive FIP [35,36,37,38]. This was been carefully investigated in a recent study that characterized T cell responses in cats with primary FIPV infection and in survivors of the primary infection that received a secondary challenge with the same virus [39]. T cell responses were not apparent during the acute phase of primary infection, but they did emerge during the secondary infection and were correlated with protection against FIPV progression. A clear message is that Type I FCoV infection does not induce a robust T cell response, regardless of whether the isolate is associated with enteric infection or FIP.

Several studies have characterized the various courses of FECV shedding (transient, intermittent, and persistent) in cats and their correlation with systemic antibody responses. A rapid decline in antibody titer after the control of FECV replication has been commonly observed [5,6,7] and was the case with the cats in this study. The lack of durability of the presumptive protective antibody response underlies the potential for repeated reinfection but also represents a challenge for the development of an FCoV vaccine. A host-pathogen balance exists between cats and FCoV such that the virus is rarely pathogenic. The mild infection does not elicit life-long immunity, thereby allowing a perpetual endemic state in cat populations with the occasional emergence of highly pathogenic FIPV. Successful immunization against FCoV will likely require induction of a robust T cell response to drive the establishment of B cell memory and longer-living plasma cells while also creating a cell-mediated immune safety net in the event of FIPV emergence [40].

It is intriguing to consider targeting FECV as a means to prevent FIPV emergence and its associated fatal disease. It seems reasonable that preventing or reducing FECV replication would reduce the chance of FIPV emergence. Anti-FECV antibodies, both systemic and mucosal, were correlated with FECV control and elimination. An orally delivered vaccine targeting protective FCoV epitopes may provide protection, particularly in the high-density and stressful environments associated with shelters and colonies. To be successful, such a vaccine would have to avoid the pitfalls of antibody-dependent enhancement that is associated with anti-spike protein IgG. The identification of protective epitopes is likely to require the mapping of cloned feline anti-FECV antibodies and the development of in vitro assays capable of testing relevant effector functions such as virus exclusion and neutralization in the context of the intestinal mucosa.

## 5. Conclusions

Virtually any finding regarding FCoV infection of cats requires a caveat. Dr. Jekyll (FECV) is really quite brilliant, causing a minimally pathogenic enteric infection that results in massive virus replication ensuring persistence of the virus in the cat population, while Mr. Hyde (FIPV) is a murderous immunopath who invariably kills once the immune response is turned against the host. It is critical to keep in mind with whom we are dealing, Dr. Jekyll or Mr. Hyde. A very different approach may be required for the prevention, diagnosis and treatment of one versus the other. Regardless, the virus remains fundamentally the same [41].

We characterized, for the first time, the mucosal humoral and cellular response to FECV and suggest that mucosal IgA and systemic IgG responses are necessary for virus control, given the lack of demonstrable cell-mediated immune responses. Significant additional work is required to characterize the viral targets and the antibody effector functions needed for successful vaccination against FCoV. A successful immunization strategy will also require the induction of a robust T cell response.

## Figures and Tables

**Table 1:** Serological status and fecal RT-PCR results for experimental groups.

Group	FCoV IgG+/Total	Fecal FCoV+/Total	Colon FCoV+/Total	Blood FCoV+/Total
1	0/18	0/18	0/18	0/18
2	6/6	0/6	0/6	0/6
3	9/9	9/9	3/9	0/9

**Table 2:** Phenotype and proliferation of colonic lymphocytes.

**Phenotype**	**Group 1**	**Group 2**	**Group 3**	**Combined**
B220+ (B cells)	11.0% ± 6.5%	7.6% ± 7.0%	9.4% ± 5.5%	10.0% ± 6.3%
CD4+	76.9% ± 8.3%	81.2% ± 10.4%	73.0% ± 13.4%	76.7% ± 10.2%
CD8+	1.6% ± 1.1%	1.3% ± 1.5%	3.8% ± 1.9%	2.2% ± 1.7%
**% Expressing Ki67**	**Group 1**	**Group 2**	**Group 3**	**Combined**
B220+ (B cells)	62.6% ± 13.7%	55.2% ± 18.4%	51.8% ± 19.7%	58.5% ± 16.5%
CD4+	20.5% ± 5.3%	18.4% ± 9.1%	19.2% ± 7.5%	19.8% ± 6.5%
CD8+	17.5% ± 14.9%	13.2% ± 9.9%	11.0% ± 13.2%	15.0% ± 13.7%

## Supplementary Materials

The following are available online at https://www.mdpi.com/1999-4915/11/10/906/s1, Table S1: Serum Chemistry and Hematology Results.
