# Supplementary material for: Mucosal Immune Response to Feline Enteric Coronavirus Infection
Source: Viruses. 2019 Sep 27;11(10):906. doi: 10.3390/v11100906 (PMC6832150; doi:10.3390/v11100906)
Supplement: Supplementary file 1 [file viruses-11-00906-s001.pdf]

Table S1. Serum Chemistry and Hematology Results.

TEST	UNITS	REFERENCE INTERVAL	GROUP 1	GROUP 2	GROUP 3
**CHEMISTRY**					
Glucose	mg/dl	68-140	74.9	79.3	89.7
BUN	mg/dl	18-35	22.9	24.7	25.1
Creat	mg/dl	0.8-2.4	1.4	1.4	1.5
Ca	mg/dl	9.2-11.1	9.8	9.6	9.6
Phos	mg/dl	3.0-6.0	4.9	4.9	4.8
Mg^{++}	mg/dl	2.0-2.7	2.6	2.6	2.5
T.Protein	G/dl	6.3-8.0	6.6	6.5	6.4
Alb	G/dl	3.1-4.4	3.9	3.7	3.7
Glob	G/dl	2.7-4.2	2.7	2.8	2.7
A/G	G/dl	0.8-1.6	1.5	1.3	1.4
Chol	mg/dl	95-270	174.3	172.3	173.1
T.Bili	mg/dl	0-0.1	0.1	0.1	0.1
Iron	mg/dl	70-175	101.8	129.0	119.7
CK	IU/L	60-350	291.6	257.0	277.4
AST	IU/L	15-45	27.2	24.3	30.3
ALT	IU/L	30-140	64.6	61.7	68.7
ALP	IU/L	10-80	27.2	19.0	19.0
GGT	IU/L	0.0-5.0	0.0	0.0	0.0
Na^{+}	mEq/L	149-157	152.4	151.8	151.9
K^{+}	mEq/L	3.7-5.4	4.5	4.4	4.5
Cl^{-}	mEq/L	114-125	114.7	114.1	115.1
$HCO3^{-}$	mEq/L	13-22	18.1	18.3	18.4
Anion Gap	mEq/L	16-26	24.2	24.0	22.7
**HEMATOLOGY**					
WBC	x10^3/ml	4.0-14.0	8.6	8.1	7.5
Hgb	G/dl	9.8-15.5	13.4	12.9	13.0
Hct	%	32-47	41.3	39.8	40.8
RBC	x10^6/ml	6.5-10.0	10.2	10.1	9.8
MCV	fl	40-52	40.8	39.7	41.8
MCHC	G/dl	32-36	32.7	32.5	32.1
Segs	x10^3/ml	2.0-12.0	3.8	3.5	3.2
Bands	x10^3/ml	0.-0.1	0	0	0
Lymphs	x10^3/ml	1.5-6.0	4.2	4.0	3.6
Monos	x10^3/ml	0-0.8	0.2	0.2	0.2
Eos	x10^3/ml	0-1.2	0.3	0.4	0.5
Basos	x10^3/ml	0-0.1	0.1	0.0	0.0
Platelets	x10^3/ml	200-500	240.9	255.2	315.2
